# Circadian Rhythm Abnormalities in Parkinson’s Disease from Humans to Flies and Back

**DOI:** 10.3390/ijms19123911

**Published:** 2018-12-06

**Authors:** Federica De Lazzari, Marco Bisaglia, Mauro Agostino Zordan, Federica Sandrelli

**Affiliations:** 1 Department of Biology, University of Padova, 35131 Padova, Italy; federica.delazzari@phd.unipd.it (F.D.L.); marco.bisaglia@unipd.it (M.B.); 2 Cognitive Neuroscience Center, University of Padova, 35100 Padova, Italy

**Keywords:** Parkison’s disease, *Drosophila*, circadian clock, dopaminergic system, PD models, *dj-1*

## Abstract

Clinical and research studies have suggested a link between Parkinson’s disease (PD) and alterations in the circadian clock. *Drosophila melanogaster* may represent a useful model to study the relationship between the circadian clock and PD. Apart from the conservation of many genes, cellular mechanisms, signaling pathways, and neuronal processes, *Drosophila* shows an organized central nervous system and well-characterized complex behavioral phenotypes. In fact, *Drosophila* has been successfully used in the dissection of the circadian system and as a model for neurodegenerative disorders, including PD. Here, we describe the fly circadian and dopaminergic systems and report recent studies which indicate the presence of circadian abnormalities in some fly PD genetic models. We discuss the use of *Drosophila* to investigate whether, in adults, the disruption of the circadian system might be causative of brain neurodegeneration. We also consider approaches using *Drosophila*, which might provide new information on the link between PD and the circadian clock. As a corollary, since PD develops its symptomatology over a large part of the organism’s lifespan and given the relatively short lifespan of fruit flies, we suggest that genetic models of PD could be used to perform lifelong screens for drug-modulators of general and/or circadian-related PD traits.

## 1. Introduction

Parkinson’s disease (PD) is a chronic, progressive neurodegenerative disorder, which is mainly characterized by motor dysfunction (i.e., bradykinesia, postural instability, muscular rigidity, and resting tremor). Motor symptoms arise from the preferential degeneration of dopaminergic neurons in the *Substantia nigra pars compacta* (SNpc), which represents one hallmark of the disease. The other specific feature of PD is the presence in the surviving neurons of cytoplasmic inclusions, referred to as Lewy bodies, which are mainly formed by ubiquitin and α-synuclein (α-syn) [1].

Besides motor dysfunctions, non-motor symptoms, such as changes in sleep, mood, cognition, and autonomic as well as visual functions, have often been observed during PD progression, and their frequency seems to be related to disease severity [2]. At least some of the non-motor symptoms are thought to be linked to neuronal alterations, as well as to decreased dopamine (DA) levels in brain areas different from the SNpc [3,4,5,6,7].

PD is mainly a sporadic disorder, the pathogenesis of which has not been fully elucidated. However, ~10% of all cases have a genetic origin, and currently at least 23 disease-segregating loci have been identified [8]. The most studied so far are the genes encoding the proteins α-syn, Parkin, PINK1, DJ-1, and LRRK2, and different animal models for these genetic forms have been generated. Although not fully reproducing all the clinical and pathological phenotypes associated with PD, these genetic models have contributed to a better definition of the cellular pathways involved in pathogenesis. These include mitochondrial dysfunction, impaired lysosomal and proteasomal degradation, deficiencies in synaptic transmission, and vesicular recycling [8].

Several lines of evidence have suggested a link between PD pathology and alterations in the circadian clock system [3,4,7], the endogenous ~24 h clock which controls the daily timing of physiological and behavioral processes in organisms. However, since PD is a complex disease, the physiological bases of the circadian alterations associated to this pathology have not been fully clarified yet. In the effort of elucidating such aspects, the possibility of performing basic and translational research studies using multiple model organisms represents a valuable approach.

In this paper, after a brief description of the circadian clock system in mammals, we will discuss a number of studies performed in humans and mammalian models, supporting the association between circadian abnormalities and PD. We will also highlight some aspects emerging from such studies which still remain elusive. Subsequently, we will focus on the insect *Drosophila melanogaster* as a possible additional model to address at least some of the open questions in investigations concerning the link between PD and the circadian clock.

## 2. Circadian Abnormalities in PD Pathology

In humans, as in other mammals, the circadian system consists of a hierarchical network of cellular clocks, localized in both the brain and peripheral organs, which are integrated at the level of the organism. The suprachiasmatic nucleus (SCN) of the hypothalamus is the central circadian clock. Light is the main environmental synchronizing stimulus of the circadian clock. The SCN receives light stimuli from the retina through the retinohypothalamic tract, and entrains the endogenous clock to the daily external environmental variations, sending signals to the peripheral clocks, and coordinating their rhythm and phase. This, in turn, determines that clock-controlled phenotypes, such as sleep/wake cycles, body temperature, hormone secretion, and metabolism are synchronized in phase with the environmental daily light-dark (LD) cycle [9].

PD patients frequently show modifications in several physiological aspects, which are fully or partly under the control of the circadian system.

### 2.1. Circadian Symptoms in PD Patients

A circadian symptom which has been detected in individuals affected by PD consists in alterations at the level of the daily rest/activity rhythm [10,11,12]. In healthy conditions, daily motor activity shows a cycling pattern, with the lowest levels during the night, and a progressive increase during the day. In PD patients, reduced amplitude in the rest/activity rhythm was recorded, with decrements in both diurnal activity and nocturnal rest [10,11,12]. In addition, it has been reported that from 65 to 95% of PD individuals suffer from sleep disturbances [13], which are now recognized as some of the major contributors to the impairment of life quality in patients [2,14,15]. Sleep dysfunctions include sleep fragmentation, excessive daytime sleepiness, and REM sleep behavior disorder (RBD), a pathology characterized by the loss of normal atonia during the REM stage of sleep. Although the causes of sleep impairments in PD are multifactorial, it is however interesting to note that RBD is considered a sleep stage under strong circadian influence [3]. 

PD may also be associated with several visual problems, such as disturbances in visual acuity, contrast sensitivity, and color vision [16]. These deficits are at least partially due to retinal DA deficiency [16], and some of them are somewhat reversible with levodopa treatment [17,18]. Interestingly, visuo-perceptual deficits have been reported in patients with RBD, with impairments in color vision and visuospatial construction supporting circadian fluctuations in visual perception. In addition, a clinical study found that contrast sensitivity in PD patients showed diurnal fluctuation that is otherwise absent in healthy people [19].

Several studies indicated that many clock-controlled parameters, such as core body temperature, blood pressure, and the daily cycling secretion of melatonin (involved in the regulation of the sleep/wake cycle) appeared to be frequently affected in PD patients [4,20,21,22,23,24,25]. Other analyses indicated altered expression levels of the cardinal human (h) clock gene, h*Bmal1* in blood samples from PD patients compared to appropriate controls [23,26] and a case-control study associated specific single nucleotide polymorphisms in h*Bmal1* and h*Per1* circadian clock genes to an increased risk of PD in a Chinese population [27]. 

Finally, it is worth mentioning that different studies reported that PD patients experienced the beneficial effects of bright light therapy (BLT), which ameliorated both non-motor and motor symptoms [3]. BLT has been successfully used to reduce depressive symptoms in several types of mood disorders and is thought to act through modulation of the circadian clock [28]. 

Taken together, these data support the association between circadian dysfunctions and PD. However, the etiology of the circadian symptoms in PD patients is not well understood. The difficulty is due to several reasons, including the fact that the pathology is extremely heterogenous, with a large and variable spectrum of symptoms [29]; the therapy used to alleviate PD can, per se, cause a variation in circadian parameters, such as the melatonin cycling phase in levodopa-treated patients [25]; some non-motor symptoms, such as sleep disturbances, and their worsening during the progression of the pathology, appear to negatively impact the circadian system of PD patients, leading to a vicious circle [30]. Studies in mammalian models have, however, generated informative data.

### 2.2. Circadian Dysfunctions in Mammalian PD Models

Both circadian and sleep phenotypes have been analyzed in several mammalian PD models, produced via genetic alterations or toxin-based treatments [3]. Here, we will highlight defects mainly associated with the circadian clock (Table 1).

Variable and age-dependent abnormalities in circadian behavioral phenotypes were detected in a mouse model overexpressing the human α-syn in the whole brain (ASO) [31], as well as in mice showing a progressive degeneration of dopaminergic neurons, due to a specific deletion in the *mitochondrial transcription factor A* gene in these neurons (MitoPark) [32] (Table 1). In addition, ASO mice displayed a reduced neuronal daytime firing rate in the SCN, although the cycling expression of the mouse (m) circadian factor mPER2 was not affected [31]. Circadian alterations were also detected in PD models induced by stereotaxic injection with toxins such as 6-hydroxydopamine (6-OHDA) [43] or 1-methyl-4-phenyl-1,2,3,6-tetrahydropyridine (MPTP) [44], both determining dopaminergic neuronal death. 6-OHDA treatments in rodents have been found to deregulate circadian behavioral and physiological outputs, as well as the expression of clock genes in different brain regions [38,39,40,41]. Furthermore, a recent study reported that levodopa administration further modified hormone secretion levels and the expression profiles of some clock genes in specific brain regions in 6-OHDA treated-rats, corroborating the notion that the pro-drug may contribute to circadian alterations seen in PD patients [42]. Contrasting data relating to the effects of MPTP treatment on mouse circadian phenotypes were obtained [33,34,35] (Table 1). However, it is interesting to note that an environmental circadian disruption (induced by a long-term exposure to a 20:4 LD cycle) exacerbated the PD-related motor deficits in this model [45]. In addition, circadian abnormalities were observed in MPTP-treated non-human primates, which showed arrhythmic behavior in constant darkness conditions (DD, when the endogenous clock free runs), indicating that DA depletion caused the loss of circadian locomotor activity, at least in this model [30]. Since MPTP treatment did not seem to influence the SCN circadian functions, the authors suggested that the behavioral defects lay downstream of the master clock [30].

Collectively, these data suggest that the circadian dysfunctions detected in PD can result from a misregulation of the complex interactions between the dopaminergic and circadian systems. Indeed, several lines of evidence indicate DA as one of the modulators of the circadian system, playing a fundamental role in the maintenance of proper rhythmicity [7,46]. DA and dopaminergic transmission seem to have relevant functions at different levels of the circadian system [3,46] as they are, for example, implicated in the modulation of the light input signals from the retina to the SCN [47], but they are also considered as output targets of the timing information from the SCN to other brain regions [3,46]. However, the cellular and molecular processes at the basis of circadian dysfunctions in PD are still unclear. In addition, further studies are required to clarify whether the circadian abnormalities are only a consequence of PD or might also represent a causal/enhancement factor during the progression of the pathology. 

Given the above considerations, studies based on the use of relatively simple and well-characterized model organisms, such as *D. melanogaster*, have the potential of providing relevant information to explore such aspects.

## 3. *Drosophila melanogaster* as a Model Organism to Study the Relationship between the Circadian Clock and PD

*D. melanogaster* is a widely accepted model organism which has been used extensively to study a range of biological and physiological phenomena, as well as several types of human pathology. Despite the evolutionary distance, many genes, cellular mechanisms, signaling pathways, as well as neuronal processes are conserved between insects and mammals. Moreover, *Drosophila* shows an organized central nervous system and well-characterized complex phenotypes, such as circadian behaviors, locomotion, sleep, memory, and learning [48]. In addition, this insect provides the advantage of a relatively easy implementation of advanced genetic techniques, which are generally more challenging to accomplish in mammals [49]. In the past years, *Drosophila* has been successfully used in the study of the circadian system and as a model for neurodegenerative disorders, including PD [50,51,52]. In the following sections we will describe the fly circadian and dopaminergic systems and we will report recent studies which indicate the presence of circadian abnormalities in some fly PD genetic models. We will also discuss the use of *Drosophila* to investigate whether the disruption of the circadian system might be causative of neurodegeneration within adult brains. Finally, we will examine further analyses and approaches which might be useful in the investigation of the link between PD and the circadian clock using *Drosophila*.

### 3.1. The Drosophila Circadian Clock

**Figure 1:**
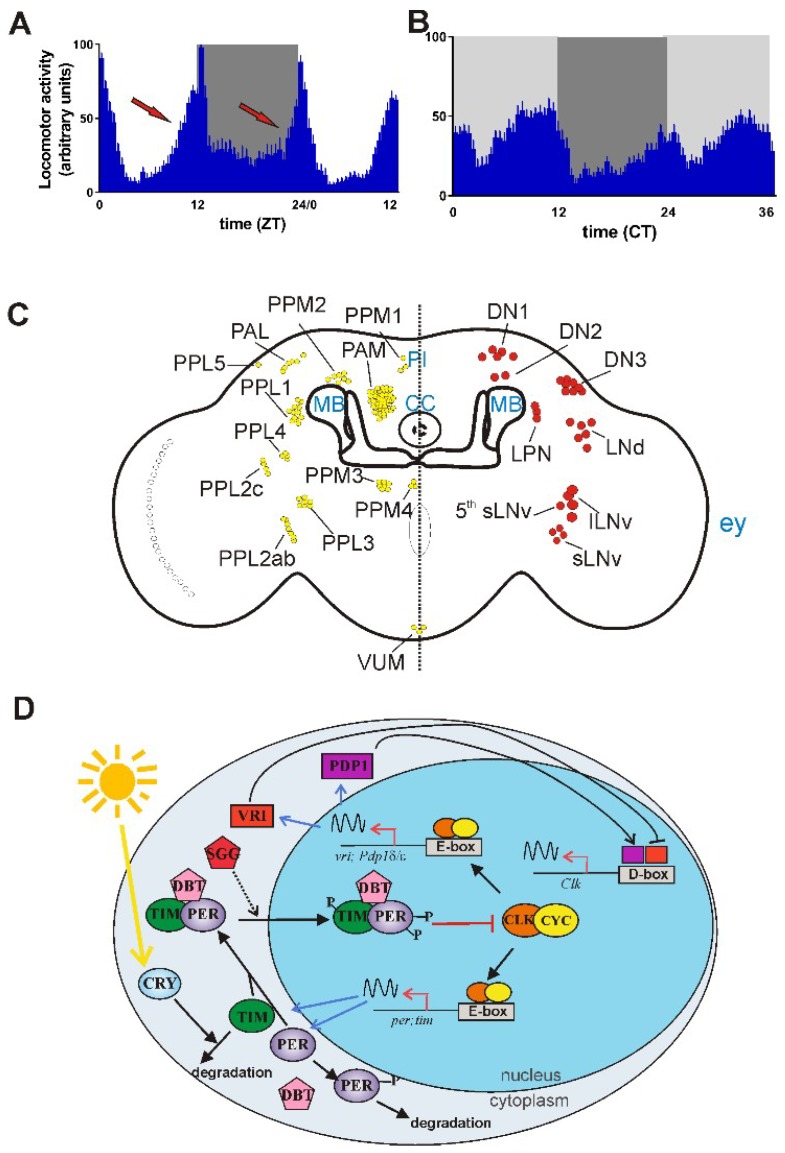
Locomotor activity and organization of the circadian clock and dopaminergic system in the brain of *D. melanogaster* (**A**,**B**) Locomotor activity (mean ± SEM) of ~50 wild-type flies in (**A**) 12:12 light-dark (LD) and (**B**) constant darkness (DD) conditions, as a function of time. In (**A**) red arrows indicate the morning and evening anticipatory activities; ZT: Zeitgeber Time. In (**B**) CT: Circadian Time; (**C**) Schematic representation of the adult fly brain, in which the relative positions of the circadian neurons (right), the dopaminergic neurons (left), the mushroom bodies (MB), the central complex (CC), the Pars Intercerebralis (PI) are reported. Right hemisphere: lLNv: large ventral lateral neurons; sLNv: small LNvs; 5th sLNv: the fifth PDF-negative sLNv; LPN: lateral posterior neuron; LNd: dorsal LNs; DN1: dorsal neurons group 1; DN2: DN group 2; DN3: DN group 3; ey: relative position of the compound eye. Left hemisphere: PAM: Protocerebral Anterior Medial group; PAL: Protocerebral Anterior Lateral group; PPM1-4: Protocerebral Posterior Medial groups 1–4; PPL1–5: Protocerebral Posterior Lateral groups 1–5; VUM: Ventral Unpaired Median group; open circles in the left optic lobe indicate the TH positive cells of the medulla. (**D**) The two major TTLs of the circadian molecular clock in *Drosophila*. In the first *Drosophila* TTL, dCLK and dCYC form a dimer which binds the Enhancer boxes (E-boxes) in the promoter of the d*per* and *dtim* clock genes. dPER and dTIM proteins interact to form a complex, enter into the nucleus, and inhibit dCLK-dCYC activity. A second TTL modulates d*Clk* expression: the dCLK-dCYC dimer induces the transcription of d*vri* and d*Pdp1 δ*/*ε* genes. dVRI and dPDP1 *δ*/*ε* compete for the same element (D-box) in the d*Clk* promoter, controlling d*Clk* transcription. Post-translational modifications mediated by the kinases dDBT and dSGG modulate clock protein activities, regulating protein–protein interactions, nuclear translocation and degradation. In some clock neurons, light activates the internal photoreceptor dCRY which associates with dTIM and mediates its degradation. CLOCK: Circadian Locomotor Output Cycles Kaput; CRY: Cryptochrome; CYC: Cycle; PER: Period; TIM: Timeless; VRI: Vrille; PDP1: Par Domain Protein 1; DBT: Doubletime; SGG: Shaggy. Black arrows indicate the flow of the process; black sinusoidal lines and red arrows indicate transcriptional activity; blue arrows indicate the protein translation process (**A**,**B**) Graphs derived from data from our laboratory; (**C**,**D**) Modified from [54].

The *Drosophila* circadian clock controls the timing of several behavioral and physiological processes, such as daily locomotor activity, eclosion, feeding behavior, temperature preferences, memory, and social behaviors [53,54,55,56]. Here, we will focus on the daily locomotor activity, since this represents the circadian behavioral output most studied in *Drosophila* PD models so far. In laboratory 12:12 LD cycles, *D. melanogaster* shows locomotor activity with a bimodal profile, characterized by a peak in the morning and a second peak in the evening. In wild-type flies, locomotor activity starts to increase slightly before the lights-on and lights-off transitions, anticipating the LD variation (Figure 1A). In DD conditions, the locomotor activity remains rhythmic, with a periodicity of ~24 h, even though, in the latter condition, the locomotor profiles show slight changes with respect to the LD profiles (Figure 1B). 

The circadian locomotor activity is driven by a master clock, located in the brain. This is formed by ~150 neurons, organized into six groups per brain hemisphere and classified as four large ventral lateral neurons (l-LNvs), five small ventral lateral neurons (s-LNvs), six dorsal lateral neurons (LNds), three lateral posterior neurons (LPNs), and dorsal neurons, subdivided into three groups (DNs 1, 2, and 3) [57] (Figure 1C). Initial analyses suggested a primary role of the four s-LNvs in controlling morning activity and of the fifth s-LNv, the LNds, and the DNs in regulating evening activity [58,59]. However, recent data indicate that the whole network of circadian clock neurons contributes to the generation of behavioral rhythmicity in both LD and DD conditions [60,61]. 

All these neurons express the fundamental clock components required to drive circadian rhythmicity at the molecular level. In a simplified model, the core of the molecular circadian clock is formed by two main interlocking autoregulatory transcriptional/translational feedback loops (TTLs) [62]. The first TTL includes the transcription factors dCLOCK (dCLK) and dCYCLE (dCYC) which form a heterodimer (dCLK/dCYC) and promote the transcription of the d*period* (d*per*) and d*timeless* (d*tim*) genes (Figure 1D). dPER and dTIM proteins are then regulated by different kinases and phosphatases at a post-translational level to modulate the timing of their rhythmic functions, their nuclear translocation, stability, and their activity as inhibitors of the dCLK/dCYC transcriptional regulators. Among the kinases, dDoubletime (dDBT) targets dPER, while dShaggy is involved in the phosphorylation of both dTIM and dPER. The dCLK/dCYC dimer also represents the positive regulator of a second TTL, which modulates the daily expression of d*Clk*. This TTL is negatively and positively regulated by dVRILLE (dVRI) and dPar Domain Protein 1 (dPDP1), respectively, which probably compete to bind the same DNA elements in the promoter region of d*Clk* [62] (Figure 1D). In mammals, the organization into TTLs of the molecular clockwork is essentially conserved, in spite of the replacement of some elements in both the first (mCryptochromes (mCRYs) instead of dTIM) and second [mRar-related orphan receptor (mROR) and mNuclear receptor subfamily 1, Group D (mREV-ERB) instead of dVRI and dPDP1] TTLs. In addition, mammals show an intrinsic redundancy, as they are characterized by the presence of different paralogs of the same molecular clock element, including three m*Per*, two m*Cry*, one m*CLK*, and one m*Neuronal PAS domain-containing protein 2* (m*Npas2*) gene. 

In flies, clock neurons show several differences in protein/neuropeptide expression profiles, properties, and activities. This variability has been associated with the roles of the different neurons in driving and fine-tuning the circadian rhythmicity at an organismal level [53,55,63,64]. A first example is linked to the capacity to perceive clock-resetting light signals. As in mammals, light represents the main cue which is responsible for the synchronization of the *Drosophila* circadian clock with the 24 h LD cycles. At the molecular level, light synchronization is mediated by the blue-light photoreceptor, dCRY [65,66]. Light activates dCRY, which interacts with dTIM, promoting its degradation [67,68] (Figure 1D). However, only a subset of clock neurons expresses dCRY [69,70,71], and the circadian clock network perceives light signals also through other pathways, including eye photoreceptors, ocelli, and the Hofbauer Buchner eyelet [72]. 

Fundamental differences are also found in the synthesis and activity of neuropeptides, such as the peptide Pigment Dispersing Factor (dPDF) [73], expressed in the l-LNvs and in 4 out of the 5 s-LNvs. dPDF acts as an output neurotransmitter, as a synchronizer of the clock neurons and in the transduction of light signals into the circadian network [57]. Other neuropeptides, including the short neuropeptide F [74] and the ion transport peptide [75], which have a role in controlling different features of the evening locomotor behavior, are expressed with variable patterns within the master clock neurons [53,55,63]. Among clock neurons, a heterogeneity in the sensitivity to several types of neurotransmitters, including glutamate, acetyilcholine, GABA, glycine, and biogenic amines [i.e., DA, serotonin, and octopamine (related to mammalian noradrenaline)], was also demonstrated and associated with different aspects of circadian locomotor behavior [53,63,64]. 

Recent studies focused on the communications between the central clock and downstream brain regions important in the generation of circadian locomotor behavior. In the current model, timekeeping signals from the master clock reach specific neurons of the *Pars Intecerebralis* (PI) (Figure 1C), which in turn contact other neurons located in the subesophageal brain region. Detailed information about these neuronal circuits are reported elsewhere [53,64,76,77,78]. 

Interestingly, it has been suggested that aminergic systems (i.e., serotoninergic, dopaminergic, and octopaminergic) also have a fundamental role as output components acting downstream from the master clock in the control of circadian locomotor rhythmicity, both in LD and DD [79]. DA was demonstrated to be important in circadian light sensitivity and entrainment, as well as in driving a robust circadian rhythmicity in free-running conditions [80]. In addition, a cooperation between at least two aminergic systems seems to be required to guarantee circadian locomotor activity both in LD and DD [79]. In fact, flies carrying a loss-of-function mutation in the vesicular monoamine transporter dVMAT, which functionally inactivates all the aminergic systems, showed an aberrant locomotor activity profile in LD and weak rhythmicity in DD [81]. On the contrary, d*Vmat^−^* flies overexpressing wild-type dVMAT in at least two out of the three systems (in all the combinations) showed a rescue of the mutant circadian phenotype in both LD and DD. Since *dVmat^−^* flies did not show evident defects in the circadian molecular oscillator within the master clock neurons, the cooperative action of the aminergic systems was postulated to act downstream of the brain master clock [79].

Finally, it has been suggested that glia-to-neuron communication also controls rhythmic behavior, since the glia modulates dPDF transport and/or release from LNv projections [82], and at this level, *ebony*, which encodes an enzyme involved in DA and histamine recycling, seems to play a key role as an output gene [83].

### 3.2. Drosophila Dopaminergic System

*Drosophila* has a well-characterized dopaminergic system which encompasses ~130 neurons, subdivided into multiple clusters which are symmetrically distributed in the fly brain [84,85,86]. The major cluster is the protocerebral anterior medial (PAM) group, which is composed of ~100 neurons, while all other groups are constituted by fewer cells (~5–10 each). These smaller clusters include the protocerebral anterior lateral (PAL), protocerebral posterior medial (PPM1–4), the protocerebral posterior lateral (PPL1–5), the Thoracic 1 (T1), and the ventral unpaired median (VUM) group (Figure 1C). While PAM, PAL, and T1 neurons are located in the anterior part of the brain, the PPM and PPL clusters are mapped posteriorly. These different groups are variably involved in the control of locomotion and other complex behaviors, including olfaction, memory, learning, courtship, reward, sleep, and arousal by communicating with organized substructures of the *Drosophila* brain, such as the Mushroom Bodies (MB) and Central Complex (CC) [84,86,87]. Moreover, small cells expressing tyrosine hydroxylase (TH) (the rate-limiting enzyme involved in DA synthesis) are located in the optic lobe at the level of the medulla neuropile [85] (Figure 1C). In addition, pathways and enzymes involved in DA synthesis, release, and signaling are conserved, although the catabolism of DA follows different mechanisms [87]. As in the case of mammals, in *Drosophila*, DA shows a rhythmic release in the form of a pronounced bimodal daily pattern with a mild increase during the end of the night in adult fly heads. This pattern seems to be influenced by light during the daytime, while it is controlled by the circadian system during the night [88]. 

DA in *Drosophila* is not just a neurotransmitter, but is required as a precursor molecule for cuticle pigmentation; therefore, *pale^−^* mutant flies lacking a functional TH present unpigmented cuticles, as well as embryonic lethality. For this reason, in order to investigate the functions of DA at the level of the brain, the main model is based on the generation of individuals that specifically preserve DA synthesis in non-neuronal cells. Interestingly, these flies displayed reduced daily activity and tended to sleep more frequently than controls; they also showed a reduced reactivity to external mechanical stimuli [89]. Remarkably, the administration of known PD drugs, such as levodopa and carbidopa, were found to rescue locomotor hypoactivity, providing evidence that with respect to these drugs, the *Drosophila* blood-brain barrier has the same permeability properties as that of mammals [90]. 

Although clock neurons do not express DA [91], and it is suggested that dopaminergic neurons do not possess a functional clock [92], different studies indicate the existence of synaptic contacts between dopaminergic and circadian-clock LNvs neurons. In particular, dopaminergic neurons appear to send input signals to l-LNv dendrites, and to receive input stimuli from s-LNv axons [93,94]. These neuronal communications contribute to regulate diurnal wakefulness in the complex interaction involved in the control of sleep and wakefulness in flies [93,94,95].

### 3.3. Drosophila PD Models and Circadian Dysfunctions

*Drosophila* is widely employed for studying the genetic forms of PD. Fly models have been generated to analyze the effects of hα-syn overexpression, as well as knockout (KO) and knockdown (KD) alterations of the *Drosophila* homologs of mammalian *Lrrk2*, *Parkin*, *PINK1*, and *DJ-1*. Recent studies have examined the circadian phenotypes in hα-syn overexpressing flies, and in d*Parkin*, d*PINK1* single mutants [96,97,98]. In the following paragraphs, we will describe the available data on these models, together with those obtained in our laboratories, analyzing circadian locomotor activity in flies with null mutations in the *Dj-1* homologs (Table 2).

#### 3.3.1. α-syn

In humans, α-syn is a small, intrinsically disordered 14 kDa protein which is abundantly expressed in neurons. Mutations and multicopy variants of its encoding gene, h*SNCA* have been associated with highly penetrant forms of PD and dominant inheritance [109]. At the neuronal level, the protein exists in an equilibrium between a highly disordered cytosolic monomer and a folded, membrane-bound form [110]. Although the physiological role is still under debate, its prevalent localization at presynaptic terminals suggests an involvement in neurotransmitter release and vesicle recycling [111]. Nevertheless, under pathological conditions, the protein conformation is thought to shift from disordered monomers to large multimeric species, forming insoluble cytosolic inclusions. Oligomers are pre-fibrillar species that are considered to be the principal culprits of hα-syn toxicity due to their potential to disrupt membrane integrity, and to alter mitochondrial functionality and protein degradation pathways [109]. Although the *Drosophila* genome does not harbor an *SNCA* homolog, different fly models expressing wild-type and mutant variants of the human gene have been generated. These models manifest a variety of aspects of human PD, including neurodegeneration, locomotor dysfunctions, and the formation of sub-cellular protein inclusions [99,112] (Table 2).

Circadian locomotor activity and sleep were analyzed in flies expressing wild-type hα-syn (wt-αS) and two mutant isoforms, A53T-αS and TP-αS, specifically in serotonergic and/or dopaminergic neurons [96]. A53T-αS (carrying a single amino acid substitution) is able to form pre-fibrillar oligomers that aggregate later than wt-αS, while TP-αS (characterized by the modification of three amino acids within the same αS molecule; Table 2) tends to oligomerize but is unable to aggregate. According to this feature, the TP-αS mutant should present a more aggressive phenotype due to its toxicity. In particular, overexpression of the wt-αS or TP-αS isoforms affected the locomotor activity profiles in 12:12 LD, since at light-off, only a mild reduction in locomotion was observed in TP-αS transgenic flies, while this locomotor reduction was clearly evident in control flies. Furthermore, in TP mutants, older individuals (30 days) did not show the typical anticipatory locomotor behavior before lights-off in LD and presented a longer circadian period in DD. This phenomenon might be related to mutant hα-syn oligomerization-induced neuronal malfunction and/or death. Given that the above described genetic manipulations occurred at the level of the aminergic system, the observed circadian behavioral alterations were probably related to effects occurring downstream from the master clock [79].

Besides circadian abnormalities, the expression of wild-type and mutant forms of hα-syn also induced alterations in the sleep profile of transgenic flies. Globally, these abnormalities were related to variations in the amount of sleep and the number and length of sleep episodes during the day or night, with such alterations appearing already in young flies, which is suggestive of neuronal dysfunction rather than neuronal death in the transgenic flies [96]. 

#### 3.3.2. Parkin and PINK1

hParkin and hPINK1 are mitochondrial-associated proteins which, when mutated, have been reported to cause autosomic recessive forms of PD. The h*PARK2* gene encodes hParkin, a cytosolic E3-ubiquitin ligase, with an amino-terminal ubiquitin-like domain and a carboxy-terminal ubiquitin ligase domain. h*PARK6* encodes the mitochondrially localized phosphatase and tensin homolog (PTEN)-induced kinase 1 (hPINK1). Although found in different subcellular compartments, the two proteins were shown (for the first time in *Drosophila*) to play a fundamental role in mitochondrial quality control [101,113,114,115]. *Drosophila* models of Parkin (in the fruit fly encoded by d*park*) and PINK1 present a shortened lifespan, male sterility, swollen mitochondria, and altered locomotor behavior, mainly as a result of muscle damage. The two proteins are thought to belong to the same pathway, with dPINK1 acting upstream of dParkin [116]. 

Experiments to evaluate the endogenous circadian rhythmicity of d*Pink1* and d*park* loss-of-function mutant flies indicate that d*Pink1* mutants show the strongest circadian locomotor activity defects in DD, when compared to d*park* null mutants. Furthermore, both mutations were associated with alterations in the electrophysiological properties of the l-LNvs, although they influenced the electrophysiological features of these clock neurons in different ways [97] (Table 2).

In addition, a recent report showed that d*Pink1* and d*park* mutant flies were characterized in LD conditions by the absence of circadian locomotor anticipatory activity in the morning (and a reduced anticipation in the evening), and more fragmented sleep compared to wild-type controls [98]. A specific downregulation of these genes mapped the circadian abnormalities at the level of the LNvs and sleep defects in the brain insulin-producing cells (IPCs), known for their role in sleep maintenance [117]. Interestingly, the morning, anticipation abnormalities were specifically due to an excessive number of endoplasmic-reticulum (ER)-mitochondrial contacts, which determined anomalous lipid trafficking and a depletion in the phosphatidylserine ER content at the level of the mutant LNvs. This alteration, in turn, caused a disruption in the physiological production of the dPDF-containing vesicles, and in the failure of proper neuropeptide localization in the LNv terminals [98]. It is noteworthy that an abnormal distribution of the circadian neuropeptide, hVasoactive Intestinal Peptide (analogous to the dPDF) was also detected in hypothalamic neurons differentiated from pluripotent stem cells of PD patients carrying mutations in h*PARK6* and h*PARK2* genes, indicating the evolutionary conservation of Pink1 and Parkin functions [98].

Cellular, circadian, and sleep defects were rescued by feeding d*Pink1* and d*park* mutant flies with phosphatidylserine, suggesting that these abnormalities were not linked to neurodegeneration, but rather to a neuronal disfunction. Moreover, the same feeding rescue results suggest that the circadian and sleep alterations were mainly associated with neuronal networks different from the dopaminergic system (i.e., LNvs and IPCs for circadian and sleep defects, respectively) [98].

#### 3.3.3. DJ-1

**Figure 2:**
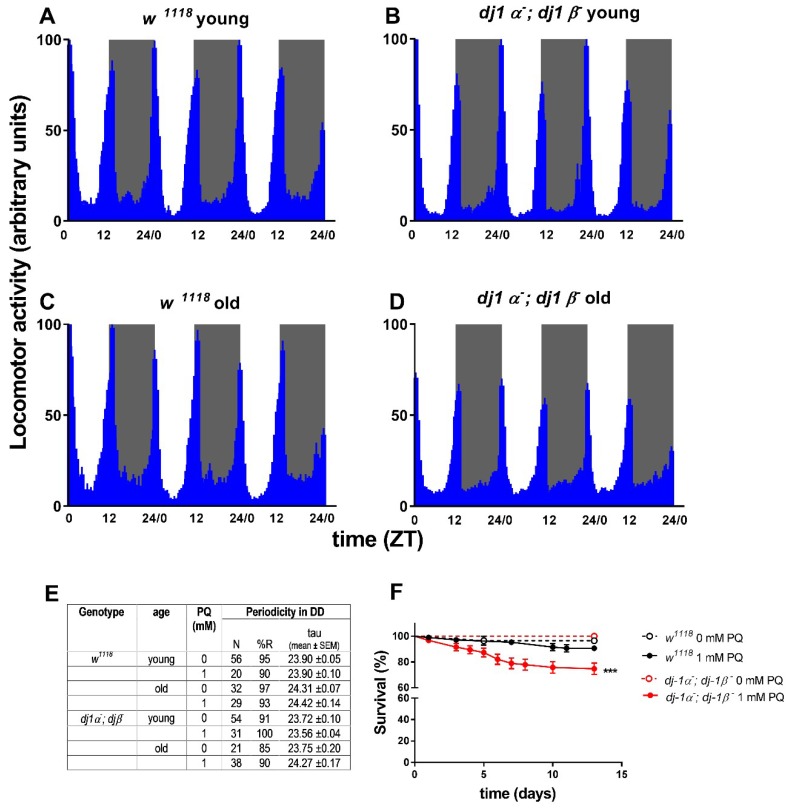
Locomotor activity in 12:12 LD and circadian periodicity in DD conditions of young (3–5-day-old) and old (30-day-old) d*dj-1α^−^*; d*dj-1β^−^* double knock-out (DKO) males. d*dj-1α^−^*; d*dj-1β^−^* DKO and *w^1118^* control flies were reared at 23 °C in 12:12 LD conditions (light intensity ~800 lux). 3–5 or ~30-day-old adult males were transferred into vials (~30 individuals each) containing normal or 1 mM Paraquat (PQ)-supplemented medium for 4 days. Subsequently, individuals were singly placed in glass tubes, containing normal or oxidative stress-inducing food. Locomotor activity was recorded as 1 min bins for 10 days in 12:12 LD conditions, or for 3 days in 12:12 LD followed by 7 days in DD regimes, using the Trikinetics Activity Monitor (DAM2). Circadian periodicity was analyzed as described in [124]. At least 3 replicates for each experiment were performed. (**A**–**D**) Locomotor activity profiles (mean ± SEM) of 50 young *w^1118^* (**A**), 61 young d*dj-1α^−^*; d*dj-1β^−^* DKO (**B**), 38 old *w^1118^* (**C**), 79 old d*dj-1α^−^*; d*dj-1β^−^* DKO (**D**) males for 3 days in 12:12 LD conditions. (**E**) Free-running periodicity of young and old d*dj-1α^−^*; d*dj-1β^−^* DKO and *w^1118^* control flies in normal or mild oxidative stress conditions (1 mM PQ). N: number of analyzed flies; %R: percentage of rhythmic flies; tau: periodicity in DD. (**F**) Survival analysis to evaluate the effectiveness of the chronic exposure to 1 mM PQ on vitality in young flies. Only PQ-treated d*dj-1α^−^*; d*dj-1β^−^* DKO males showed a significant decrease in vitality (***: *p* < 0.0001 for all paired comparisons in Mantel-Cox test; for each genotype and treatment at least 90 individuals were monitored in 3 independent replicates).

In humans, mutations in *DJ-1* have been associated with early-onset forms of PD, with recessive inheritance. hDJ-1 is a small dimeric protein, with ubiquitous expression and a conserved amino acid sequence from Prokaryotes to Eukaryotes. The protein is mainly cytosolic, but a small fraction has also been detected within mitochondria [118,119] and in the nucleus [120]. Although the physiological role of hDJ-1 is still controversial, many activities have been ascribed to the protein, including oncogenesis, antioxidative responses, male fertility, and transcription [121]. Nonetheless, the most corroborated function is its involvement in anti-oxidant responses, activating pro-survival pathways while inhibiting cell death [121]. Differently from mammals, *Drosophila*’s genome encodes two *DJ-1* paralogs, d*dj-1α* and d*dj-1β*. While d*dj-1α* is principally transcribed in the male testes [107] and partly in the brain [122], d*dj-1β*, similarly to the human protein, is ubiquitously expressed [107]. Double knock-out (DKO) flies are viable and fertile and are characterized by a normal lifespan, but show sensitivity to oxidative insults determined by chronic exposure to 20 mM Paraquat (PQ). In particular, dDj-1*β* seems to be primarily involved in oxidative stress protection, since *dj-1β^−^* single mutants display sensitivity to oxidative stressors similarly to d*dj-1α^−^*; d*dj-1β^−^* DKO flies [107]. In our laboratories, we investigated the consequences of d*dj-1s* loss on locomotor activity profiles in 12:12 LD, and circadian rhythmicity in DD. As PD is an age-related pathology, young (3–5-day-old) and old (30-day-old) flies were analyzed. Both young and old individuals exhibited a normal locomotor activity profile in LD, with both anticipatory morning and evening activity (Figure 2A–D). No particular abnormalities were detected in the circadian periodicity of neither young nor old d*dj-1α^−^* and d*dj-1β^−^* DKO flies in DD conditions (Figure 2E). Circadian locomotor behavior of d*dj-1α^−^* and d*dj-1β^−^* DKO flies was not even affected under a mild oxidative-stress treatment, consisting of chronic exposure to a low concentration of PQ (1 mM). Similar treatments are known to cause arrhythmicity in *foxo^−^* mutant flies, a genetic situation which is known to produce an oxidative stress-sensitized background [123]. In our case, 1 mM PQ chronic exposure was able to determine a slight, but significant, decrease in vitality in d*dj-1α^−^* and d*dj-1β^−^* DKO flies, indicating the effectiveness of the oxidative-stress treatment (Figure 2F). However, neither the percentage of rhythmic flies nor the periodicity were significantly modified under this oxidative-stress condition (Figure 2E). Since DJ-1 is known to have a role in the protection against oxidative stress, our data suggest that this factor does not influence the physiological mechanisms controlling rhythmic behavior in flies. 

## 4. *Drosophila* as a Model to Evaluate the Effect of Circadian Disruptions on Neurodegenerative Processes

An interesting aspect which requires further investigation is whether circadian clock disruptions can promote/enhance neurodegenerative processes, in this way representing an additional risk factor for the development of PD pathology. Indeed, it is currently accepted that an impairment in normal rhythmicity favors the activation of gene expression and pro-inflammatory responses, which may play a role also in neurodegeneration. A proposed pathway that associates the master clock to neurodegeneration is through the modulation of oxidative stress responses. However other mechanisms such as neuroinflammation or an impaired degradation of pathogenic protein aggregates might also be involved [125]. 

Studies in mice showed that the KO of m*Bmal1* or the DKO of m*Clk* and its paralog m*Npas2*, promoted premature aging, increased oxidative stress, age-dependent neuropathology as well as synaptic degeneration, and indicated these factors as possible players in the anti-oxidant response, through a downstream activation of antioxidant response transcription factors [126,127,128]. In addition, as mentioned above, it was demonstrated that an environmental disruption of the circadian clock intensified PD-related motor deficits in MPTP-treated mice. These abnormalities were associated with a loss of dopaminergic neurons and a robust neuroinflammatory reaction in the *Substantia Nigra* [45]. Similarly, in 6-OHDA treated mice, mutations of the circadian clock element mREV-ERBα (the m*Bmal1* transcriptional repressor) promoted 6-OHDA-dependent locomotor deficits, dopaminergic neurodegeneration, and neuroinflammation in the vertebral midbrain [129].

Compelling evidence indicates that *D. melanogaster* represents a valuable model to investigate these aspects. In flies the oxidative stress response is under circadian control, as wild-type individuals exposed to an oxidative insult during the day time were more sensitive than those treated at night. Moreover, flies with a disrupted circadian clock, as a result of either maintaining wild-type flies under constant light (LL) conditions, or by using clock mutants such as *per^0^*, did not show daily fluctuations in this response [130]. In fact, aged *per^0^* mutant flies displayed higher impaired locomotor ability, increased susceptibility to oxidative damage, and expanded vacuolization in the central brain. Furthermore, the absence of functional dPER exacerbated damage in neurodegeneration-prone mutants, suggesting that in flies this clock gene acts as a neuroprotective factor during aging [131,132]. Moreover, in flies a long term arrythmicity is able to determine an acceleration in the manifestation of phenotypes typical of the aging process, since wild-type flies in LL conditions as well as amorphic (*per^0^*, *tim^0^* or *cyc^0^*) or hypomorphic (*Clk^AR^*) clock mutants showed a significantly reduced lifespan and more pronounced locomotor impairments compared to their age-related controls [131,133]. 

In *Drosophila*, a connection between clock molecular elements, circadian neuronal cells and neurodegeneration was recently demonstrated by two different groups [133,134]. 

In particular, within the circadian neuronal network, Means and colleagues identified a molecular pathway involving the circadian kinase dDBT, the tetratricopeptide repeat-containing protein dSpaghetti (dSpag), and the dDronc caspase, implicated in the cleavage of the tubulin-associated unit (dTau) protein [134]. Tau is an evolutionarily conserved protein, which, in the truncated form, shows enhanced aggregation, contributing to the neuronal death typical of human neurodegenerative diseases, including Alzheimer’s disease [135] and, albeit to a lesser extent, PD [136]. Following an extensive series of experiments, and based on the use of mutant and transgenic flies, the authors obtained compelling indications to the effect that under normal conditions, dSpag controls the stability of dDBT, which in turn inhibits the activation of dDronc. On the contrary, following perturbations which determine the degradation of dDBT (for example via a d*spag* KD), dDronc is activated, leading to an enhanced assembly of truncated dTau (and likely other still unknown targets), which in turn contributes to neuronal death [134].

The same authors also demonstrated that a d*spag* KD, driven specifically in clock neurons, determined the activation of dDronc in regions outside the circadian network (i.e., optic lobe). dDronc activation was evident when flies were exposed to light and modulated by dPDF signaling, indicating a light-mediated communication between circadian neurons and other brain regions, leading to a modulation of the activity of molecular factors involved in apoptosis [134]. Similar abnormal activation of dDronc was also detected in young flies expressing a mutated form of dDBT (DBT^K/R^) [137] in the circadian network, in d*Clk* null mutant individuals (*Clk ^Jrk^*) and in very old wild-type flies, which are characterized by alterations in dDBT metabolism. These data suggest the dSpag-dDBT-dDronc pathway as being one of the possible molecular mechanisms by means of which a disrupted clock might affect the state of health and vitality of neuronal cells.

Evidence for a connection between the circadian clock and neurodegeneration more specifically involving the dopaminergic system was derived from the work by Vaccaro and coauthors [133]. In this study, the authors demonstrated that, in flies, the core clock element d*Clk* showed additional clock-independent activity within the s-LNvs, which was required to avoid locomotor impairments typical of *Clk^AR^* hypomorphic mutants. Experiments performed by specifically knocking down or expressing d*Clk* within s-LNvs in a *Clk^AR^* mutant background indicated that this function was, however, linked to dPDF-mediated communication and protected against the neurodegeneration of the PPL1 dopaminergic neurons, conceivably through the control of apoptotic phenomena, possibly acting via paracrine signaling between the two systems [133]. However, the communication might also be synaptic, since synaptic connections between axons of the sLNvs and dopaminergic neurons were recently described, although the specific dopaminergic cluster/s involved in this connection has not yet been clearly identified [94]. These data revealed a functional communication between the circadian clock network and the dopaminergic systems in *Drosophila*, and implied a direct involvement of a circadian clock element in the maintenance of healthy dopaminergic neurons during aging in flies. Interestingly, an age-dependent neuroinflammatory phenotype, with astrogliosis and synaptic terminal degeneration, was detected in the brain cortex of mice following the simultaneous deletion of m*Clk* and its paralog m*Npas2* [127]. However, in this case, the presence of specific degeneration in dopaminergic neurons was not investigated. 

## 5. Conclusions and Further Perspectives

**Figure 3:**
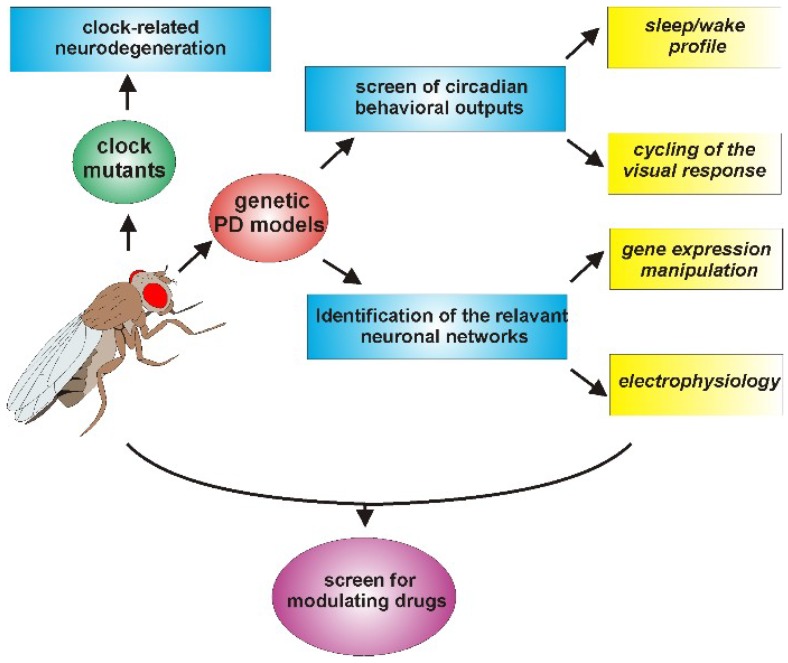
Evaluation of the relationship between PD and circadian clock alterations using *D. melanogaster*. Clock gene mutants (green) can be used to determine the possible presence of clock-related neurodegeneration, while circadian abnormalities might be evaluated in genetic PD models (red), at the behavioral, physiological, and molecular levels. Similar analyses could be performed to screen for modulating drugs (magenta), in order to ameliorate circadian alterations.

Although the number of studies concerning the relationship between PD and the circadian clock performed so far using *Drosophila* is still limited, the results obtained are, in our opinion, promising, particularly in view of considering this organism as an additional model to explore these aspects (Figure 3).

The use of circadian locomotor activity as a first-level behavioral output has indicated that some of the genes implicated in the early-onset forms of PD (e.g., h*SNCA*, d*Pink1*, and d*park*) were able to affect the circadian rhythmicity in *Drosophila*, but others, such as the d*dj-1s*, were not. Given the multifactorial etiology of PD, globally, these data suggest that the genetic factors involved in the pathology might not control the same phenotypes, and an initial screen in *Drosophila* could provide a useful indication to guide subsequent analyses in more complex organisms. 

In addition, the relative ease with which downregulation or overexpression of candidate genes in specific neurons can be obtained in flies allows to address the question of which, among the different types of neurons, are mainly involved in affecting the circadian output, as seen in the case of hα-syn in aminergic neurons [96] and d*Pink1* or d*park* in LNv neurons [98]. This strategy could contribute to elucidate the causal factors of the circadian abnormalities in PD, as demonstrated by Valadas and colleagues who associated the d*Pink1^−^* and d*park^−^* circadian defects mainly to alterations in the neuropeptide vesicle formation at the level of the circadian neurons [98]. The same approach might therefore be extended to other PD-related genes evaluating whether they directly affect the molecular and/or the electrophysiological properties of the circadian neurons, or whether they act through the dopaminergic system or other circuits, which in turn modify the activity of the clock neuronal network. 

Moreover, besides locomotor activity, other phenotypes show a circadian pattern in *Drosophila*. Among these is the optomotor response [138,139], the study of which might constitute a useful phenotypic output for the symptoms related to visual acuity and contrast sensitivity detected in some PD patients and fly PD models [16,140,141]. The study of these circadian phenotypes in *Drosophila* PD models (including the manipulation of the expression of PD-related gene products in specific sets of cells and/or neurons) might contribute to the identification of the cellular network/s, the malfunction of which might determine such vision-related defects in flies. Despite the differences in the interactions between the visual and the circadian systems in insects and mammals, this kind of information might be relevant to the clarification of the mechanisms underlying the presence of circadian alterations also in individuals affected by idiopathic forms of PD. Generally, dopaminergic neurons are considered to be the primary target in the explanation of the motor symptoms observed in PD patients. However, non-motor symptoms, including some circadian abnormalities, might originate from the “spreading” of alterations to other (among which are possibly also non-dopaminergic) neurons [142,143].

Data obtained from neurodegeneration studies in flies support the notion that *Drosophila* might be an additional model to evaluate the impact of a misregulated or disrupted circadian clock in the neurodegenerative processes. Given the differences in the structure and organization of the circadian and dopaminergic systems between flies and mammals, it is likely that not all the information might be directly transposable from one model to the other. For example, it is important to mention that, while in flies, d*per* seems to have a role as a neuroprotective factor in aging brains [131,132], in mice, the DKO of the paralogs m*Per1* and m*Per2* did not cause any neuroinflammatory phenotype [127]. In such DKO mice, we can speculate that the presence of a third *Per* gene (m*Per3*) might (partially) substitute for the lack of m*Per1* and m*Per2* in the putative neuroprotective function. However, no data exploring this aspect are yet available. It is worth underlining that when data obtained from both models are consistent, the use of *Drosophila* allows to more readily perform experiments which both take longer and are usually more complex in mammals. For example, tissue-specific rescue experiments in mutant backgrounds, which can be conducted with relative ease in *Drosophila*, allow to rapidly explore multiple experimental conditions in order to understand the cellular and molecular mechanisms involved in the control of a specific phenotype, including neurodegeneration.

Furthermore, because PD, like most neurodegenerative diseases, progresses over a period of decades, pharmacological interventions that even slightly modulate one or more of the pathways involved in the pathogenesis of the disease could delay the progression or improve function, leading to an enhancement in the quality of life for patients suffering from such terribly debilitating disorders. In this respect, the various available *Drosophila melanogaster* genetic models of PD could provide an advantage as first-stage screens for modulatory drugs (as seen in [98]). In particular, the relatively short lifespan of fruit flies facilitates the search for modulators of PD, which develops its symptomatology over periods of time often spanning a large part of the organism’s lifespan. In this respect, in *Drosophila*, it is feasible to conduct lifelong studies during which it would be possible to explore the pharmacological modulation, for instance, of perturbations in the circadian rhythms resulting from the manipulation of genetic determinants of PD, such as hα-syn, and the homologs of the human Pink1, Parkin, or LRRK2. 

An aspect that has been considered partly in PD models of *Drosophila* so far is sleep, which has been examined in d*Pink1* and d*park* mutants [98] and in flies overexpressing hα-syn, either by characterizing sleep profiles under basal conditions [96] or by evaluating the effects of sleep deprivation on memory [144]. In *Drosophila* and mammals, sleep is controlled by both circadian and homeostatic systems and, as mentioned above, is often affected in PD patients. Under this perspective, it is interesting to remind that, using the appropriate sampling frequency, the same raw data collected for the analysis of daily locomotor behavior in flies could be processed to determine the structure of sleep [145,146]. This would therefore allow to evaluate the presence of sleep abnormalities in the different genetic PD-related forms, and possibly to understand the neuronal circuit/s mainly affected in *Drosophila*, as well as to evaluate the rescue of altered sleep patterns as an end-point in primary screens for pharmacological interventions.

## Figures and Tables

**Table 1:** Circadian dysfunctions in mammalian Parkinson’s disease (PD) models.

Mammalian Models	Intervention	Animal Model	Circadian Phenotype
ASO	Overexpression of wt α-syn in all brain regions	Mouse	Fragmented circadian rhythms and a reduced firing rate of SCN neurons during the day [31]
MitoPark	Deletion for *mitochondrial transcription factor A* in dopaminergic neurons	Mouse	Age-dependent rhythm decline and disturbed circadian activity rhythms under constant high light conditions [32]
MPTP-injection	Intraperitoneally and subcutaneously [33] Intraperitoneally [34]	Mouse	No significant changes in circadian parameters [33,34]
	Not specified [35]	Mouse	Reduced amplitude in locomotor rhythm; altered clock gene expression in the SCN [35]
	Intravenously [36]	Dog	Circadian urine volume and vasopressin release alteration [36]
	Intramuscular [37]	Non-human Primates	Loss of circadian locomotor activity in the absence of light/dark cues [37]
6-OHDA-injection	Bilateral in the *striatum* [38]	Rat	Altered clock gene expression in the *striatum* [38]
	Intracerebroventricular and unilateral infusion in medial forebrain bundle [39]	Rat	Disorganized wheel-running pattern in constant darkness and blunted *PER2* expression rise in the dorsal *striatum* [39]
	Bilateral in the ventral tegmental area [40]	Rat	Reduced locomotor activity period in LD and longer activity rhythm periodicity under constant dim light [40]
	Bilateral in the *striatum* [41]	Rat	Decreased amplitude of the heart rate rhythm [41]
	Bilateral in the *striatum* [42]	Rat	Altered clock gene expression profile in SNC and in the *striatum* [42]

**Table 2:** *Drosophila* genetic PD models and associated circadian abnormalities.

Fly Genetic PD Model					
Gene	Genetic Manipulation	PD Phenotype			Circadian Phenotype
		Dopaminergic Cells Death	Protein Inclusions	Locomotor Deficit	
h*SNCA*	wt*-αS* overexpression	Yes [99]	No [99]	Yes [99]	Altered locomotor activity profiles [96]
	*A53T* overexpression	Yes [99]	Yes [99]	Yes [99]	n.d
	*TP-αS* (A30P, A56P, A76P) overexpression	Yes [96]	Yes [100]	Yes [100]	Circadian locomotor periodicity shift with aging [96]
d*Pink1*	Loss of function	Yes [101]	n.d.	Yes [101]	Arrhythmic, hyperexcitability of l-LNvs neurons, day/night difference in RMP less pronounced [97]
					Absence of circadian locomotor anticipatory activity in the morning in LD [98]
d*park*	Loss of function	Yes [102]	n.d.	Yes [102]	Weakly rhythmic, No difference in day/night SFR ratio [97]
					Absence of circadian locomotor anticipatory activity in the morning in LD [98]
d*dj-1α*	Loss of function	Yes [103]	n.d.	n.d.	n.d.
d*dj-1β*	Loss of function	No [104]	No [105]	Yes [105,106]	n.d.
d*dj-1α;* d*dj-1β*	Loss of function	No [107]	n.d.	Yes [108]	No evident abnormalities observed (this work)

n.d: not determined; RMP: Resting Membrane Potential; SFR: Spontaneous Firing Rate.
